# Impact of a 9-1-1-Integrated Mobile App on Bystander CPR: Implementation of PulsePoint in an Urban County

**DOI:** 10.3390/jcm15010005

**Published:** 2025-12-19

**Authors:** Charles W. Hwang, Anthony J. Meyer, Ira Harmon, Brandon P. Climenhage, Eric M. Nordhues, Torben K. Becker

**Affiliations:** 1 College of Medicine, University of Florida, Gainesville, FL 32610, USA; bclimenhage@ufl.edu; 2 Department of Emergency Medicine, College of Medicine, University of Florida, Gainesville, FL 32610, USAt.becker@ufl.edu (T.K.B.); 3 Center for Data Solutions, College of Medicine, University of Florida, 655 W 8th St., Jacksonville, FL 32209, USA

**Keywords:** out-of-hospital cardiac arrest, emergency medical services, mobile applications

## Abstract

**Background/Objectives:** Early bystander CPR helps to restore perfusion and improves the likelihood of favorable survival and neurological outcome after out-of-hospital cardiac arrest (OHCA). One strategy to improve bystander CPR is the use of crowd-sourcing mobile CPR applications such as PulsePoint, which notifies bystanders of nearby OHCA. In 2019, PulsePoint was deployed in an urban county. Prior to its deployment, bystander CPR rates were 42.9% in this county. This descriptive analysis seeks to analyze bystander intervention after PulsePoint implementation in an urban county. **Methods:** This retrospective observational study included all PulsePoint activations in Alachua County from June 2020 to September 2023. Patient characteristics and survey data were extracted from EMS patient care reports, hospital electronic medical records, and Pulsepoint dispatch and responder data. Descriptive statistics were used to analyze patient and responder characteristics, PulsePoint activation circumstances, and patient care. **Results:** Of 225 PulsePoint activations, 95 (42.2%) were confirmed OHCA. Among these, 54 (56.8%) received bystander CPR prior to EMS arrival. Out of 15 prehospital defibrillations, laypersons defibrillated 9 patients (60.0%). There was an average of 3.3 eligible PulsePoint responders within a 0.25-mile radius of the OHCA victim. A majority of PulsePoint survey respondents were formally trained in CPR and automated defibrillator use. **Conclusions:** The data from our urban EMS experience demonstrate that bystander CPR rates were higher after PulsePoint deployment (56.8%) than before. Our bystander CPR rate was also higher than the national average.

## 1. Introduction

Out-of-hospital cardiac arrest (OHCA) is a major cause of morbidity and mortality in the US. The American Heart Association (AHA) estimates 356,000 OHCA cases occur annually in the US [1].

The likelihood of survival after OHCA is highest when cardiopulmonary resuscitation (CPR) and defibrillation is initiated within 3 to 5 min after cardiac arrest [2]. After cardiac arrest, the probability of neurologically intact survival decreases by 7–10% for every minute without CPR; with early CPR, this decline may be reduced to as little as 3% per minute [3,4]. Moreover, early defibrillation increases the likelihood of restoring a perfusing rhythm [5]. Therefore, early, timely CPR after OHCA is crucial for survival.

The average EMS response interval after OHCA is approximately 8 min [6]. Consequently, the role of bystanders has been recognized as an essential link in the Chain of Survival for OHCA resuscitation. Bystander intervention can shave critical minutes from the initiation of life-saving interventions and improve the likelihood of return of spontaneous circulation (ROSC) and survival [5].

Public awareness of OHCA and engagement in CPR are essential for favorable outcomes. One innovative public health mechanism to improve community involvement is through smartphone mobile applications designed to notify the lay public of nearby OHCAs. These applications, also known as crowdsourcing bystander CPR mobile applications for OHCA, have resulted in significant increases in bystander CPR frequency and survival outcomes in Europe [2,7]. Similar technological initiatives have been implemented in the US, one of which is PulsePoint (PulsePoint Foundation, Pleasanton, CA, USA).

PulsePoint is a mobile application for users who have indicated they are trained in CPR and willing to assist in an emergency. The application uses GPS location services to alert nearby users of a potential OHCA contemporaneous with EMS dispatch and directs the users to the precise location. PulsePoint also maintains an automated external defibrillator (AED) registry and provides directions to the nearest AED. PulsePoint enables CPR-trained citizens to respond to OHCAs, thus reducing collapse-to-CPR and collapse-to-defibrillation intervals [8].

From the initial release of PulsePoint in 2011 to January 2020, there were 350,000 requests for assistance from nearby responders for 100,000 OHCA events across the US. PulsePoint was rolled out in Alachua County, Florida by University of Florida College of Medicine Emergency Medicine faculty in 2019. Prior to 2019, bystander CPR rates for OHCA in Alachua County were 42.9%. Here, using the Standards for Quality Improvement Reporting Excellence (SQUIRE) 2.0 guidelines [9], we present a descriptive analysis of bystander OHCA response after PulsePoint implementation in Alachua County.

## 2. Materials and Methods

### 2.1. Setting

This was a retrospective observational review conducted in Alachua County, located in north-central Florida. Alachua County is an urban county that encompasses 2266 km^2^ (875 mi^2^) with a population of 278,468 (2020 census). The city of Gainesville is the largest city in Alachua County; it is 167 km^2^ (64.5 mi^2^) with a population of 141,085 (2020 census).

Alachua County Fire Rescue (ACFR) is the sole transport agency within the county and delivers advanced life support (ALS) level of care. At the time of the study, ACFR staffed 12 full-time ALS ambulances and 4 critical care ambulances that responded to approximately 50,600 requests for emergency medical care annually and transported approximately 26,200 patients annually to local emergency departments. Non-transport first response is provided by ACFR (ALS) and several municipal departments (ALS or basic life support [BLS]). All EMS agencies within the county are fire department-based EMS systems.

During the period of this analysis, Alachua County had two teaching hospitals and a Veteran Affairs Medical Center that offered a complete spectrum of medical services, including post-arrest care and percutaneous coronary intervention (PCI) capabilities. While there are several free-standing emergency departments (FSEDs) in Alachua County, OHCA patients are not transported to FSEDs per medical protocols unless extenuating circumstances arise.

### 2.2. Project Design and Patient Population

We performed an analysis of all patients who had a PulsePoint dispatch issued by the Alachua County public-safety answering point (PSAP) between 1 June 2020 and 30 September 2023. Reviewed records included EMS electronic patient care reports (ePCRs) from ACFR (ESO^®^, Austin, TX, USA), available hospital electronic medical records (EMRs) (Epic^©^ version 100.2512.0.0, Epic Systems, Madison, WI, USA), and PulsePoint dispatch and responder survey data.

All PulsePoint dispatches issued by the Alachua County PSAP between June 2020 and September 2023 were included. Exclusion criteria included PulsePoint dispatches without a corresponding EMS ePCR (“no chart”) or without a corresponding bona fide patient (“no patient”). PulsePoint dispatches that were canceled by first response (i.e., law enforcement or EMS) were included for descriptive statistics but not included in the analysis between OHCA and non-OHCA cohorts. All procedures were performed in compliance with relevant laws and institutional guidelines. This project was deemed a quality improvement project by the University of Florida Institutional Review Board and was thus not subject to its ongoing oversight.

### 2.3. Aim

The primary goal of this project was to provide a descriptive analysis of bystander OHCA response after PulsePoint implementation in Alachua County. Secondary goals were to present PulsePoint responder survey responses.

### 2.4. Measurements

Dispatch determinant code and emergency medical dispatch (EMD) complaint; patient location using GPS coordinates; number of eligible PulsePoint responders (within 0.25-mile radius) and AEDs in the vicinity (within 0.25-mile radius); GPS coordinates of all eligible PulsePoint responders; actual times that the 9-1-1 call was received and when EMS and PulsePoint were dispatched were collected from PulsePoint and the prehospital ePCR. Patient age, sex, and Utstein-style variables were also abstracted from the ePCR. Hospital EMRs, if available, were reviewed to assess whether ROSC was achieved in the hospital. Distances and approximate driving times were estimated using Google Maps.

Two authors (CWH, AJM) independently reviewed the ePCR narrative and the attending paramedic’s “Primary Impression” to determine whether the patient experienced a true OHCA event. Discrepancies were resolved by discussion and consensus decision; if it remained unclear whether the patient experienced a true OHCA event after discussion, the encounter was classified as a true event.

All eligible PulsePoint responders receive a voluntary, standardized survey (Appendix A) from PulsePoint after each incident. The survey responses were provided by PulsePoint Foundation for further analysis.

### 2.5. Data Analysis

Descriptive statistics were used to analyze patient and responder characteristics, PulsePoint activation circumstances, and patient care. The Fisher Exact test was used to determine statistical significance for categorical variables. The Wilcoxon Rank Sum test was used to assess statistical significance for continuous variables. Odds ratios with confidence intervals and *p*-values are reported. Two-sided *p*-values of <0.05 were considered statistically significant. Statistical analyses were performed using R version 4.3.2.

## 3. Results

### 3.1. General Characteristics

**Figure 1:**
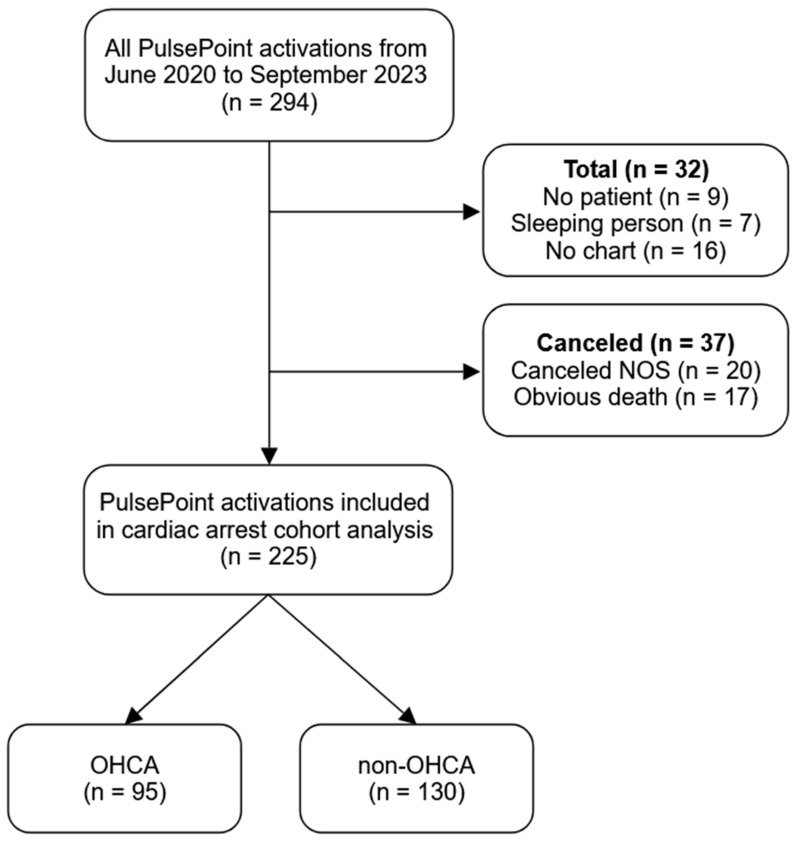
Selection of the study cohort. Abbreviations: NOS, not otherwise specified; OHCA, out-of-hospital cardiac arrest.

Of the 294 PulsePoint activations during the study period, 32 activations were excluded because of the reasons listed in Figure 1. An additional 37 activations were cancelled by first response. Of the remaining 225 activations, 95 (42.2%) patients were determined to have likely experienced true OHCA, while 130 (57.8%) patients were classified as non-OHCA (Table 1). There were several instances (*n* = 7) where CPR was started but the patient regained consciousness prior to EMS arrival; because CPR was started, these cases were characterized as true OHCA events. Specifically, ROSC was obtained before EMS arrival at doctor’s offices and dialysis centers (*n* = 5), including one incident where a patient became unresponsive and had “bradycardia of 20”; after bystander CPR (*n* = 1); and after law enforcement CPR (*n* = 1).

Patients with true OHCA were generally older than those with non-OHCA (67 [IQR: 47–78] years vs. 43 [IQR: 25–64] years, *p* < 0.001, respectively). Respiratory-related EMD complaints and dispatch determinant codes (e.g., abnormal breathing, ineffective breathing, etc.) were consistently found to represent non-OHCA events. In the non-OHCA cohort, overdose, syncope, seizure, altered mental status, and alcohol intoxication represented 82 (63.1%) cases.

Bystander CPR was initiated in more than half of true OHCA events (*n* = 54, 56.8%) by both health care professionals (*n* = 30, 55.6%) and other laypersons (*n* = 24, 44.4%). 37 (38.9%) patients with true OHCA were located at a health care facility. On average, there were 3.3 eligible PulsePoint responders and 2.2 AEDs within a 0.25-mile radius of the victim.

The most common cardiac arrest rhythms were non-shockable (*n* = 67, 70.5%) and shockable (*n* = 10, 10.5%). The initial rhythms for 3 OHCA cases were characterized as sinus rhythm. These represented situations where CPR was initiated either at a physician’s office or dialysis center but the patients regained consciousness and had sinus rhythm prior to EMS arrival; although the paramedic’s primary impression was either syncope or respiratory failure, these cases were characterized as true OHCA because CPR was initiated.

PulsePoint and EMS dispatch occurred relatively contemporaneously within 11 s of each other. CPR initiation occurred a median of 1 min prior to EMS dispatch, while it took EMS a median of 6 (IQR: 5–8) minutes to arrive on scene. Defibrillation was performed by EMS (*n* = 6, 40.0%) and other laypersons (*n* = 9, 60.0%).

### 3.2. Survey Results

Of the 294 PulsePoint activations during the study period, 128 survey responses were obtained for 86 (29.3%) unique PulsePoint activations (Table 2). Among survey respondents, there were, on average, 6.1 eligible PulsePoint responders within a 0.25-mile radius. On average, the survey responders were within 0.29 miles by car for an average approximate driving time of 1.78 min.

Although OHCA represented 69 (80.2%) dispatch determinant codes and 56 (65.1%) EMD complaints of the 86 incidents with available survey results, only 29 (33.7%) of the 86 incidents were true OHCA events. Survey respondents represented 20 (23.3%) cases involving bystander CPR.

Nearly all respondents (*n* = 121, 94.5%) were aware that a PulsePoint dispatch had been issued. Most respondents (*n* = 96, 75.0%) were health professionals (i.e., hospital or prehospital staff) with formal CPR and AED training (Supplementary Materials). 48 respondents (37.5%) made their way towards the emergency, while 20 (41.7%) arrived and found the patient in need of medical attention. Respondents cited being unavailable (*n* = 17), EMS personnel already on scene (*n* = 15), and not seeing the notification (*n* = 7) as common reasons for not responding.

Upon arrival, 10 respondents encountered a patient requiring CPR or a deceased patient, while 6 respondents encountered a non-OHCA patient requiring medical attention. 7 respondents actively provided CPR, 4 provided rescue breaths, and 4 used an AED.

## 4. Discussion

Cardiac Arrest Registry to Enhance Survival (CARES) data reported by the AHA highlight the importance of bystander CPR. Bystander CPR is associated with improved survival-to-hospital discharge (11.2% vs. 7.0%) and survival with favorable neurological function (9.5% vs. 5.4%) [10]. In 2022, there were 147,736 non-traumatic EMS-treated OHCAs reported to CARES by EMS agencies [11]. Of these, bystander CPR was performed on 40% of patients, while 11% received bystander AED use [11].

Early defibrillation increases the likelihood of restoring a perfusing rhythm and prevents deterioration into asystole [5]. Even if an AED is unavailable, bystander CPR alone can improve successful defibrillation upon EMS arrival. However, if an AED is available and used by a bystander in conjunction with early CPR, up to 70% of OHCA victims may survive with good neurologic outcomes [5]. Although non-shockable rhythms are most common in OHCA, by arriving earlier, bystanders may potentially encounter more shockable rhythms, initiate life-saving interventions sooner, and improve the likelihood of favorable outcomes.

Mobile applications, like PulsePoint, have this exact goal of early bystander arrival to improve time-to-intervention. In Denmark, one of these applications resulted in bystanders arriving before EMS 42% of the time [2].

The findings from our retrospective descriptive analysis are similar to OHCA rates found elsewhere [12,13,14]. Our study revealed that 42% of dispatches were true OHCAs, of which approximately 25% were obvious deaths confirmed by EMS. Bystanders initiated CPR in more than half (56.8%) of true OHCAs, an increase from 42.9% prior to PulsePoint implementation. If obvious death cases are excluded, bystander CPR was initiated in 79.4% of OHCA cases. Both figures represent a significantly higher bystander intervention rate as compared to that presented by CARES, underscoring a meaningful improvement in community response following PulsePoint implementation and highlighting a major strength of this study.

The sample size was small, preventing the identification of any meaningful true versus non-OHCA predictors. However, many medical complaints, such as choking, overdose, convulsions, and breathing problems were not represented in the true OHCA cohort. There was also a statistically significant difference in age between the true and non-OHCA cohorts.

Interestingly, 61.1% of dispatches determined to be true OHCA were at public locations, which is atypical with mobile applications. One study from Baltimore referenced only 7.6% of their dispatches to the public domain [8]. In our cohort, a large proportion (68.5%) of bystander CPR initiation occurred at health care facilities, limiting its generalizability.

Encouragingly, of the registered PulsePoint users that provided survey responses, many had formal CPR (85.5%) and AED (77.3%) training. Moreover, there were, on average, 3.3 eligible PulsePoint users in a quarter-mile radius of the patient, which could greatly improve time-to-intervention, as EMS dispatch to arrival took a median of 6 (IQR:5,8) minutes. Unfortunately, however, the survey data demonstrated systematic and gradual attrition during the Chain of Survival paradigm; the true scope of this attrition is unclear due to the limitations of the PulsePoint survey logic, missing data, and incomplete survey response.

While bystander CPR is associated with improved ROSC and neurologically intact survival, its deployment can be limited by multiple social and situational barriers. During the COVID-19 pandemic, bystander CPR rates declined, likely due to infection exposure risk and social distancing concerns [15]. Female gender is less likely to receive bystander CPR [16]. Prior survey data reveals additional barriers include legal concerns, misunderstanding of Good Samaritan laws, uncertainty on recognizing cardiac arrest, lack of CPR training or confidence in one’s skills, and fear of causing harm. Social and environmental factors, such as unsafe settings and uncertainty of whether others would stop to assist can also dissuade bystanders from performing CPR [17]. Similar barriers were seen within our survey data as well. These limitations highlight the need for system-level strategies, including continued public outreach and education, to reduce hesitation, facilitate community engagement, and increase rapid bystander action.

This analysis reveals important information regarding the utility of crowdsourcing CPR mobile applications and their ability to increase community engagement for OHCA. The AHA’s position recognizes the potential for digital strategies to improve OHCA patient outcomes and emphasizes the need for rigorous research to prove safety and efficacy [18]. In addition, it has highlighted bystander CPR as a key target, with a goal of increasing bystander CPR to greater than 50% by 2030 nationwide [19].

Globally, other communities have implemented similar forms of digital crowdsourcing to increase bystander CPR. In 2015, the Singapore Civil Defense Force launched the myResponder app, which alerts CPR-trained users of potential OHCAs within 400 m of their location [20]. In 2015, researchers performed a randomized controlled trial on Stockholm’s mobile phone positioning and dispatch system, which demonstrated significantly higher rates of bystander CPR with its use, although clinical outcomes displayed no significant change [21]. In 2020, KATRETTER, another bystander CPR-crowdsourcing application, was launched in Berlin. Of 16,505 KATRETTER activations in its first two years, bystander CPR was performed in 1195 (7.2%) cases [22].

A 2016 survey of 1274 PulsePoint users who received a notification found that 79% of those who arrived at an OHCA before EMS performed bystander CPR [3]. Public opinion for PulsePoint also reflects widespread community support, with greater than 96% of North American survey responses in favor of PulsePoint implementation within their local communities [23].

### Limitations

There are several limitations to our report. As a retrospective observational analysis, biases potentially exist due to its retrospective nature, missing data, survey logic, and survey responses. The clinical uncertainty of specific timepoints and prehospital and hospital unknowns results in missing datapoints that must be inferred from various sources. Consequently, time measurements are difficult to ascertain, and time intervals are difficult to calculate.

Similarly, initial rhythm was only available if clearly reported by the responding EMS team. This alludes to the limitation of recall bias inherent to studies with survey data. An example of this can be seen in the eight cases that were counted as an arrest but may have been syncope or hypotension. While a larger sample size might demonstrate a statistically significant association between bystander CPR and ROSC, it would not improve these aforementioned time and rhythm limitations.

Alachua County is an urban county with several large tertiary-care receiving hospitals and is served by high-performance EMS agencies that regularly encounter cardiac arrest. These factors limit this analysis’ generalizability. There are areas within Alachua County that are more sparsely populated; these relatively rural regions may have fewer bystanders available but also have fewer EMS units readily available to respond to requests for service. Future studies utilizing geographic information systems (GIS) to compare bystander intervention and EMS response patterns across different geographic areas would provide valuable insights into spatial disparities in cardiac arrest care.

Similarly, 31% of bystander responders for true OHCA were healthcare professionals. Although this reflects the demographic of our community with several large hospitals, more importantly, this reflects the significance of community engagement and involvement in OHCA care. Without PulsePoint, these healthcare professionals would be unaware of nearby OHCA, and OHCA victims would be dependent on traditional 911 EMS response. With PulsePoint, these healthcare professionals are integrated into the PulsePoint dispatch paradigm, enabling highly trained individuals to respond expeditiously.

Finally, with recorded data showing defibrillation occurring in a 60:40 bystander to EMS ratio, precisely how early bystander intervention is impacting the chain of survival is difficult to measure.

As crowdsourcing mobile CPR applications become more widespread and integrated within the cardiac arrest Chain of Survival, additional data will help to elucidate the true impact of bystander CPR on OHCA outcomes.

## 5. Conclusions

OHCA is a common condition that is time-sensitive and requires urgent resuscitation. Early CPR and defibrillation help to restore perfusion and improve the likelihood of favorable survival and neurological outcome. One strategy to improve time-to-intervention is the use of crowd-sourcing mobile CPR applications such as PulsePoint. The data from our urban EMS experience demonstrate that PulsePoint implementation was associated with substantially higher bystander CPR rates, emphasizing its importance in cardiac arrest care. Future studies can further characterize geospatial variability in bystander response and identify areas for targeted community intervention. Other areas of study include identifying prehospital predictors of true cardiac arrest.

## Figures and Tables

**Table 1:** General characteristics of all patients for whom PulsePoint was activated.

	All Patients (*n* = 225)	True OHCA (*n* = 95)	Non OHCA (*n* = 130)	*p*-Value ^1^
**Dispatch**				
EMD Complaint, n (%)				<0.001
Cardiac Arrest	155 (68.9%)	90 (94.7%)	65 (50.0%)	
Choking	21 (9.3%)	-	21 (16.2%)	
Unconscious/Fainting	11 (4.9%)	2 (2.1%)	9 (6.9%)	
Overdose/Poisoning/Ingestion	8 (3.6%)	-	8 (6.2%)	
Sick Person	7 (3.1%)	1 (1.1%)	6 (4.6%)	
Convulsions/Seizure	7 (3.1%)	-	7 (5.4%)	
Breathing Problem	4 (1.8%)	-	4 (3.1%)	
Altered Mental Status	2 (0.9%)	-	2 (1.5%)	
Unknown Problem/Person Down	2 (0.9%)	-	2 (1.5%)	
Other	8 (3.6%) ^2^	2 (2.1%) ^3^	6 (4.6%) ^4^	
Determinant Code, n (%)				<0.001
SCA—Generic Sudden Cardiac Arrest	185 (82.2%)	92 (96.8%)	93 (71.5%)	
11D01—Abnormal breathing (Partial obstruction)	15 (6.7%)	-	15 (11.5%)	
11E01—Complete obstruction/Ineffective breathing	7 (3.1%)	-	7 (5.4%)	
11D01—Not Alert	2 (0.9%)	-	2 (1.5%)	
09D01—Ineffective Breathing	1 (0.4%)	-	1 (0.8%)	
12D01—Not breathing	1 (0.4%)	-	1 (0.8%)	
14E01—Arrest (out of water)	1 (0.4%)	1 (1.1%)	-	
Other	13 (5.8%)	2 (2.1%)	11 (8.5%)	
**Patient demographic and clinical data**				
Age, years, median (IQR)	52 (30, 73)	67 (47, 78)	43 (25, 64)	<0.001
Unknown age, n (%)	14 (6.2%)	7 (7.4%)	7 (5.4%)	
Sex, n (%)				0.028
Male	128 (56.9%)	62 (65.3%)	66 (50.8%)	
Female	84 (37.3%)	26 (27.4%)	58 (44.6%)	
Unknown	13 (5.8%)	7 (7.4%)	6 (4.6%)	
Public Location, n (%)				0.011
Yes	179 (79.6%)	68 (71.6%)	111 (85.4%)	
No	46 (20.4%)	27 (28.4%)	19 (14.6%)	
Patient located at Healthcare Facility, n (%)	61 (27.1%)	37 (38.9%)	24 (18.5%)	<0.001
Bystander CPR, n (%)	66 (29.3%)	54 (56.8%)	12 (9.2%)	<0.001
Healthcare Professional	32 (14.2%)	30 (31.6%)	2 (1.5%)	
Bystander/Layperson	17 (7.6%)	13 (13.7%)	4 (3.1%)	
Friend/Family/Roommate	10 (4.4%)	4 (4.2%)	6 (4.6%)	
Law Enforcement/Security	5 (2.2%)	5 (5.3%)	-	
First Responder	2 (0.9%)	2 (2.1%)	-	
No/Unknown	159 (70.7%)	41 (43.2%)	118 (90.8%)	
Initial Heart Rhythm, n (%)				<0.001
Not specified/Not obtained	98 (43.6%)	15 (15.8%)	83 (63.8%)	
Cardiac Arrest (non-shockable, e.g., asystole/PEA)	67 (29.8%)	67 (70.5%)	-	
Cardiac Arrest (shockable, e.g., VF/VT/AED)	10 (4.4%)	10 (10.5%)	-	
Sinus rhythm (e.g., NSR, tachycardia, BBB, etc.)	50 (22.2%)	3 (3.2%)	47 (36.2%)	
EMS Clinician’s Primary Impression/Presumed Cause (top causes) ^5^, n (%)				<0.001
Cardiac Arrest	60 (26.7%)	60 (63.2%)	-	
Overdose	34 (15.1%)	1 (1.1%)	33 (25.4%)	
Obvious Death ^6^	27 (12.0%)	27 (28.4%)	-	
Syncope/Fainting	22 (9.8%)	2 (2.1%)	20 (15.4%)	
Seizure	12 (5.3%)	-	12 (9.2%)	
Altered Mental Status	9 (4.0%)	-	9 (6.9%)	
Alcohol Intoxication/Use	8 (3.6%)	-	8 (6.2%)	
Foreign body in respiratory tract	5 (2.2%)	-	5 (3.8%)	
Sleeping	4 (1.8%)	-	4 (3.1%)	
Respiratory Failure	4 (1.8%)	1 (1.1%)	3 (2.3%)	
Drowning	2 (0.9%)	2 (2.1%)	-	
Traumatic circulatory arrest	1 (0.4%)	1 (1.1%)	-	
Allergic reaction	1 (0.4%)	1 (1.1%)	-	
**PulsePoint Response**				
Eligible PulsePoint responders in 0.25-mile radius, mean (min, max)	3.3 (0, 75)	3.3 (0, 61)	3.2 (0, 75)	0.273
None available, n (%)	2 (0.9%)	1 (1.1%)	1 (0.8%)	
AEDs in 0.25-mile radius, mean (min, max)	2.2 (0, 83)	2.7 (0, 83)	1.9 (0, 29)	0.043
None available, n (%)	141 (62.7%)	69 (72.6%)	72 (55.4%)	
**Process**				
Resuscitation Attempted prior to ED (i.e., bystander, PulsePoint, EMS), n (%)				<0.001
Yes	69 (30.7%)	69 (72.6%)	-	
No	25 (11.1%)	25 (26.3%)	-	
N/A	130 (57.8%)	-	130 (100.0%)	
Do Not Resuscitate	1 (0.4%)	1 (1.1%)	-	
Time Intervals				
911 Call to PulsePoint dispatch, secs, median (IQR)	79 (54, 114)	64 (49, 90)	89 (61, 123)	
PulsePoint Dispatch until EMS dispatch, secs, median (IQR)	11 (−2, 46)	1 (−5, 21)	31 (1, 59)	
EMS Dispatch to CPR initiation, min, median (IQR)	−1 (−2, 4)	−1 (−2, 4)	-	
Unknown, n	46	35	11	
EMS Dispatch to EMS on scene, min, median (IQR)	6 (5, 8)	6 (4, 7)	7 (5, 9)	
EMS Dispatch to first defibrillation, min, median (IQR)	9 (5, 23)	9 (5, 23)	-	
EMS, n (%)	6 (2.7%)	6 (6.3%)	-	
Bystander, n (%)	5 (2.2%)	5 (5.3%)	-	
LEO/First Responder, n (%)	2 (0.9%)	2 (2.1%)	-	
Healthcare Professional/Doctor’s Office, n (%)	2 (0.9%)	2 (2.1%)	-	
Unknown if defibrillated (BLS First Response arrived first), n (%)	5 (2.2%)	5 (5.3%)	-	
Patient Disposition, n (%)				<0.001
UF Health Adult ED	100 (44.4%)	26 (27.4%)	74 (56.9%)	
Deceased (including DNR)	46 (20.4%)	46 (48.4%)	-	
HCA North Florida Hospital	41 (18.2%)	18 (18.9%)	23 (17.7%)	
UF Health Pediatric ED	17 (7.6%)	5 (5.3%)	12 (9.2%)	
Other ED (FSED, VAMC)	3 (1.3%)	-	3 (2.3%)	
Other (No EMS needed, no transport, Refusal, Tele911)	18 (8.0%)	-	18 (13.8%)	
**Outcomes (only for true cardiac arrest, *n* = 95)**				
Sustained ROSC in prehospital, n (%)				
No	-	77 (81.1%)	-	
Yes	-	18 (18.9%)	-	
Transported to hospital in OHCA, n (%)	-	31 (32.6%)	-	
ROSC in the ED, n (%)				
Unknown	-	5 (16.1%)	-	
No	-	21 (67.7%)	-	
Yes	-	5 (16.1%)	-	

^1^ Fisher’s exact test; Wilcoxon rank sum test. ^2^ Allergic Reaction/Stings; Chest Pain; Diabetic Problem; Drowning/Diving/SCUBA Accident; Heart Problems; Hemorrhage/Laceration; Stroke/CVA; Traffic Accident. ^3^ Drowning; Traffic Accident. ^4^ Stroke/CVA; Diabetic Problem; Heart Problem; Chest pain; Hemorrhage/Laceration; Allergic Reaction. ^5^ Full list included in Supplemental Material. ^6^ Obvious death that was not canceled prior to Advanced Life Support arrival. Abbreviations: AED, automated external defibrillator; BBB, bundle branch block; BLS, basic life support; CPR, cardiopulmonary resuscitation; DNR, do not resuscitate; ED, emergency department; EMD, emergency medical dispatch; EMS, emergency medical services; FSED, freestanding emergency department; HCA, Hospital Corporation of America; LEO, law enforcement officer; NSR, normal sinus rhythm; OHCA, out-of-hospital cardiac arrest; PEA, pulseless electrical activity; ROSC, return of spontaneous circulation; UF, University of Florida; VAMC, Veterans Affairs Medical Center; VF, ventricular fibrillation; VT, ventricular tachycardia.

**Table 2:** General characteristics for those patients (*n* = 86) for whom PulsePoint was activated, surveys were distributed to nearby PulsePoint responders, and survey responses were received from PulsePoint responders; inclusive of both out-of-hospital cardiac arrest and non-out-of-hospital cardiac arrest cohorts.

	All Patients (*n* = 86)
**Dispatch**	
EMD Complaint, n (%)	
Cardiac Arrest	56 (65.1%)
Choking	7 (8.1%)
Unconscious/Fainting	4 (4.7%)
Overdose/Poisoning/Ingestion	4 (4.7%)
Sick Person	2 (2.3%)
Breathing Problem	2 (2.3%)
Unknown Problem/Person Down	2 (2.3%)
Convulsions/Seizure	1 (1.2%)
Other (heart problem, MVC, canceled, no chart)	8 (9.3%)
Determinant Code, n (%)	
SCA—Generic Sudden Cardiac Arrest	69 (80.2%)
11D01—Abnormal breathing (Partial obstruction)	5 (5.8%)
11E01—Complete obstruction/Ineffective breathing	2 (2.3%)
09D01—Ineffective Breathing	1 (1.2%)
11D02—Not Alert	1 (1.2%)
Other	8 (9.3%)
Eligible PulsePoint responders in 0.25-mile radius, mean (min, max)	6.1 (1, 75)
AEDs in 0.25-mile radius, mean (min, max)	5.1 (0, 83)
None available, n (%)	42 (48.8%)
Google Maps distance, miles, mean (min, max)	0.29 (0.00, 1.50)
Driving time, minutes, mean (min, max)	1.78 (1, 6)
**Patient demographic and clinical data**	
Age, years, median (IQR)	44 (33, 70)
Unknown age, n (%)	25 (29.1%)
Sex, n (%)	
Male	34 (39.5%)
Female	28 (32.6%)
Other (unknown, canceled, no patient, no chart)	24 (27.9%)
Public Location, n (%)	
Yes	72 (83.7%)
No	14 (16.3%)
Patient located at Healthcare Facility, n (%)	12 (14.0%)
Bystander CPR, n (%)	20 (23.3%)
Healthcare Professional	9 (10.5%)
Bystander/Layperson	8 (9.3%)
Friend/Family/Roommate	1 (1.2%)
Law Enforcement/Security	1 (1.2%)
First Responder	1 (1.2%)
No/Unknown/Canceled/No patient	66 (76.7%)
True Cardiac Arrest, n (%)	
Yes	29 (33.7%)
No	42 (48.8%)
Unknown/Unclear	4 (4.7%)
No chart/No patient/Canceled	11 (12.8%)
EMS Clinician’s Primary Impression/Presumed Cause (top causes), n (%)	
Cardiac Arrest	17 (19.8%)
Overdose	12 (14.0%)
Obvious Death ^1^	11 (12.8%)
Canceled/No patient/No chart	11 (12.8%)
Syncope/Fainting	6 (7.0%)
Alcohol Intoxication/Use	5 (5.8%)
Sleeping	4 (4.7%)
Seizure	3 (3.5%)
Altered Mental Status	3 (3.5%)
Foreign body in respiratory tract	2 (2.3%)
Unconscious	2 (2.3%)
Respiratory Distress/Disorder	2 (2.3%)
No complaints	2 (2.3%)
Drowning	1 (1.2%)
Traumatic circulatory arrest	1 (1.2%)
Allergic reaction	1 (1.2%)
Hypoglycemia	1 (1.2%)
Suicide Attempt	1 (1.2%)
Respiratory Arrest	1 (1.2%)
Patient Disposition, n (%)	
Hospital ED	49 (60.0%)
Deceased (including DNR)	18 (20.9%)
Other (No EMS needed, no transport, Refusal, Tele911)	5 (5.8%)
Canceled/No record/No patient	14 (16.3%)

^1^ Obvious death that was not canceled prior to Advanced Life Support arrival. Abbreviations: AED, automated external defibrillator; CPR, cardiopulmonary resuscitation; DNR, do not resuscitate; ED, emergency department; EMD, emergency medical dispatch; EMS, emergency medical services; MVC, motor vehicle collision.

## Data Availability

All data are available upon request from the corresponding author.

## Supplementary Materials

The following supporting information can be downloaded at: https://www.mdpi.com/article/10.3390/jcm15010005/s1, Table S1: Survey data.

## Abbreviations

The following abbreviations are used in this manuscript:

ACFR	Alachua County Fire Rescue
AED	automated external defibrillator
AHA	American Heart Association
ALS	advanced life support
BLS	basic life support
CARES	Cardiac Arrest Registry to Enhance Survival
CPR	cardiopulmonary resuscitation
EMD	emergency medical dispatch
EMR	electronic medical record
EMS	emergency medical services
ePCR	electronic patient care reports
FSED	freestanding emergency department
OHCA	out-of-hospital cardiac arrest
PCI	percutaneous coronary intervention
ROSC	return of spontaneous circulation

## Appendix A

Voluntary PulsePoint survey issued to every PulsePoint user that has been dispatched to an out-of-hospital cardiac arrest.

Are you aware that PulsePoint recently sent you a CPR-needed alert?
  - Yes
  - No

Select the response that best describes why you might have missed the recent PulsePoint alert.
  - I was in a noisy environment that could have resulted in me not hearing the alert
  - I was apart from my phone for a period of time and may not have been present for the alert
  - My phone was in mute/silent/do not disturb mode or set to very low volume
  - I don’t believe I received the alert

Select the response that best describes your profession.
  - EMS Provider (e.g., Paramedic, EMT)
  - Firefighter, Firefighter Paramedic or other fire department personnel
  - Physician, Physician Assistant, Medical Student/Resident
  - Other health care professional
  - Nurse, Nurse Practitioner, Nursing Student
  - Police Officer, Deputy Sheriff, or other law enforcement personnel
  - Other (please specify)

Were you on-duty at the time AND dispatched to the incident independent of PulsePoint?
  - Yes
  - No

Select the response that best describes any CPR training you have received.
  - Formal CPR training course
  - Hands-only CPR training
  - No CPR training

Have you received any training on using an automated external defibrillator (AED)?
  - Yes
  - No

Did you react to the alert by making your way towards the location of the emergency indicated by PulsePoint?
  - Yes
  - No—Why not? (choose best answer)
    EMS personnel were already there, or I heard/saw that they were responding
    The location was a facility where I thought other trained medical staff would respond (e.g., hospital, medical office, skilled nursing facility, etc.)
    I was unavailable to respond (e.g., working and unable to leave, childcare, sleeping, etc.)
    I didn’t see the alert in time to respond (e.g., didn’t hear the alert, didn’t have my phone, etc.)
    I didn’t feel adequately trained/prepared
    The emergency location was too far away
    The address information was incomplete, or I didn’t know how to get to the location provided in the alert
    Other (please briefly describe)

Did you arrive at the location AND find the person in need of medical attention?
  - Yes
  - No—Why not? (choose best answer)
    EMS personnel were already there, or I heard/saw that they were responding
    The location was a facility where I thought other trained medical staff would respond (e.g., hospital, medical office, skilled nursing facility, etc.)
    I was unavailable to respond (e.g., working and unable to leave, childcare, sleeping, etc.)
    I didn’t see the alert in time to respond (e.g., didn’t hear the alert, didn’t have my phone, etc.)
    I didn’t feel adequately trained/prepared
    The emergency location was too far away
    The address information was incomplete, or I didn’t know how to get to the location provided in the alert
    Other (please briefly describe)

When you arrived at the location, what was your assessment of the person in need of medical attention? (choose best answer)
  - I saw professional rescuers arriving and decided to turn away (e.g., ambulance personnel, fire department personnel)
  - I could not access the location because of some physical barrier (e.g., locked door, security, refusal of building entry)
  - The location was a facility where I thought other trained medical staff would respond (e.g., hospital, medical office, skilled nursing facility, etc.)
  - I couldn’t find the location or the person needing CPR (e.g., location too vague, incorrect, missing, etc.)
  - The alert was removed/cleared from my device prior to my arrival
  - I received an app notification that my help was no longer required
  - The call was determined not be a cardiac arrest and no CPR was required

Did you perform chest compressions (pushing on the chest) on the person?
  - Yes
  - No—Why not?

Did you perform rescue breathing on the person (e.g., mouth-to-mouth resuscitation, artificial ventilations)?
  - Yes
  - No—Why not?

Did you attempt to locate a nearby automated external defibrillator (AED) (i.e., search yourself, ask others to search for you)?
  - Yes
  - No—Why didn’t you search for an AED?

Did you use the AED location information in the PulsePoint app in an attempt to locate a nearby AED?
  - Yes
  - No—Why not?

Did you use an automated external defibrillator (AED) on the person?
  - Yes
  - No—Why not?

It is possible to experience psychological distress when helping with a cardiac arrest. What psychological impact did the experience have on you?
How old are you?
What is your gender?
