# Supplementary material for: Impact of a 9-1-1-Integrated Mobile App on Bystander CPR: Implementation of PulsePoint in an Urban County
Source: J Clin Med. 2025 Dec 19;15(1):5. doi: 10.3390/jcm15010005 (PMC12786782; doi:10.3390/jcm15010005)
Supplement: Supplementary file 1 [file jcm-15-00005-s001.zip › jcm-4002294-supplementary.pdf]

	Total respondents (n = 128)										
**Q1**: Were you aware that PulsePoint sent you an alert?											
A: Yes, n (%)	121 (94.5%)										
A: No, n (%)	7 (5.5%)										
A: noisy environment, n (%)	2 (28.6%)										
A: apart from phone, n (%)	2 (28.6%)										
A: silent mode, n (%)	1 (14.3%)	**Q2**: Were you on-duty at the time AND dispatched to the incident independent of PulsePoint?		**Q3**: Describe the CPR training you have received.			**Q4**: Have you received training on using an AED?	**Q5**: What psychological impact did the experience have on you?		**Q6**: How old are you? (years)	**Q7**: Male gender[2]
A: did not receive, n (%)	2 (28.6%)										
**Profession**	n (%)	A: Yes, n (%)	A: No, n (%)	A: Formal CPR training, n (%)	A: Hands-only CPR training, n (%)	A: No CPR training, n (%)	A: Yes, n (%)	A: Mildly affected[1], n	A: Do not feel affected[1], n	A: age range multiple choice (n)	n
Firefighter, firefighter paramedic, other fire department personnel	31 (24.2%)	14 (45.2%)	17 (55.8%)	31 (100.0%)	0 (0%)	0 (0%)	30[3] (96.8%)				
EMS provider (e.g., paramedic, EMT)	30 (23.4%)	5 (16.7%)	25 (83.3%)	30 (100.0%)	0 (0%)	0 (0%)	30 (100.0%)	1	1	<19 (1) 20-29 (2)	3
Police officer, deputy sheriff, other law enforcement personnel	2 (1.6%)	0 (0%)	2 (100.0%)	2 (100.0%)	0 (0%)	0 (0%)	2 (100.0%)				
Nurse, nurse practitioner, nursing student	18 (14.1%)			18 (100.0%)	0 (0%)	0 (0%)	18 (100.0%)				
Physician, physician assistant, medical student/resident	12 (9.4%)			11 (91.7%)	1 (8.3%)	0 (0%)	11[3] (91.7%)		1	20-29 (1) 30-39 (1) >70 (1)	3
Other health care professional	5 (3.9%)			no data	no data	no data	no data			20-29 (1)	1
Unknown	11 (8.6%)			no data	no data	no data	no data				
Other[4]	19 (14.8%)			17 (89.5%)	1 (5.3%)	1 (5.3%)	19 (100.0%)	1		<19 (1) 30-39 (1)	2

[1] missing data; all available data was tabulated

[2] survey respondents were exclusively male

[3] missing one datapoint

[4] Civilian logistics officer (1), Clinical research coordinator (1), Community health worker (3), Education (1), EMS Division Chief (1), FF/EMT student (2), Former FF/EMT (1), Healthcare communications (1), Hospital marketing person who promotes PulsePoint to the community (1), Lifeguard (1), Normal person (1), Retired USAF officer/EMT trained (1), Security officer (1), Undergraduate student (3)

Table S1A. PulsePoint survey responses from June 2020 to September 2023. Because of the voluntary nature of the survey and the design of the survey form, the dataset is incomplete (especially for questions 5-7); all available data have been tabulated.

		**Q8**: Did you make your way towards the location of the emergency indicated by PulsePoint?	**Q9**: Did you arrive at the location AND find the person in need of medical attention?	**Q10**: When you arrived at the location, what was your assessment of the person in need of medical attention?						**Q11**: Did you perform CPR on the person?	**Q12**: Did you perform rescue breaths (e.g., mouth-to-mouth, artificial ventilation)?	**Q13**: Did you attempt to locate a nearby AED (e.g., search yourself, ask others to search)?	**Q14**: Did you use the PulsePoint app to locate a nearby AED?	**Q15**: Did you use an AED on the patient?
**Profession**	n (%)	A:<br>Yes / No / Unknown,<br>n (%)	A:<br>Yes / No / Unknown,<br>n (%)	A: The person needed medical attention, but not in cardiac arrest (e.g. seizure, syncope, etc.)	A: The person was unconscious and not breathing normally	A: The person appeared to have overdosed and required CPR	A: The person was already deceased when I arrived	A: The person was not in need of medical attention when I arrived (e.g. no longer choking, intoxicated, etc.)	A: I was unable to assess the person (for whatever reason)	A: Yes / No, n	A: Yes / No, n	A: Yes / No, n	A: Yes / No, n	A: Yes / No, n
Firefighter, firefighter paramedic, other fire department personnel	31 (24.2%)	Yes: 11 / 31 (35.5%)<br>No: 6 / 31 (19.4%)<br>Unkn: 14 / 31 (45.2%)	Yes: 2 / 11 (18.2%)<br>No: 8 / 11 (72.7%)<br>Unkn: 1 / 11 (9.1%)				1	1						
EMS provider (e.g., paramedic, EMT)	30 (23.4%)	Yes: 10 / 30 (33.3%)<br>No: 15 / 30 (50.0%)<br>Unkn: 5 / 30 (16.7%)	Yes: 5 / 10 (50.0%)<br>No: 5 / 10 (50.0%)	1	2	1	1			Yes: 2<br>No: 1	Yes: 1<br>No: 2	Yes: 1<br>No: 2	No: 1	Yes: 1
Police officer, deputy sheriff, other law enforcement personnel	2 (1.6%)	Yes: 0 / 2 (0%)<br>No: 2 /2 (100.0%)<br>Unkn: 0 / 2 (0%)	N/A											
Nurse, nurse practitioner, nursing student	18 (14.1%)	Yes: 4 / 18 (22.2%)<br>No: 14 / 18 (77.8%)<br>Unkn: 0 / 18 (0%)	Yes: 1 / 4 (25.0%)<br>No: 3 / 4 (75.0%)	1										
Physician, physician assistant, medical student/resident	12 (9.4%)	Yes: 10 / 12 (83.3%)<br>No: 2 / 12 (16.7%)<br>Unkn: 0 / 12 (0%)	Yes: 6 / 10 (60.0%)<br>No: 4 / 10 (40.0%)	4	2					Yes: 2<br>No: 1	Yes: 2<br>No: 1	No: 3 (AED already available x3)		Yes: 2<br>No: 1
Other health care professional	5 (3.9%)	Yes: 3 /5 (60.0%)<br>No: 2 / 5 (40.0%)<br>Unkn: 0 / 5 (0%)	Yes: 3 / 3 (100.0%)<br>No: 0 / 3 (0%)		1			1	1	Yes: 1	Yes: 1	Yes: 1	Yes: 1	Yes: 1
Unknown	11 (8.6%)	no data	no data	no data	no data	no data	no data	no data	no data	no data	no data	no data		
Other[1]	19 (14.8%)	Yes: 10 / 19 (52.6%)<br>No: 8 / 19 (42.1%)<br>Unkn: 1 / 19 (5.3%)	Yes: 3 / 10 (30.0%)<br>No: 7 / 10 (70.0%)	1	2					Yes: 2	No: 2	No: 2 (AED already available x1)		No: 1
Notes:		Why not?<br>I was unavailable to respond (17)<br>EMS personnel were already there or I heard/saw that they were responding (15)<br>I didn't see the alert in time to respond (7)<br>The location was a facility where I thought other trained medical staff would respond (3)<br>The emergency location was too far away (4)	Why not?<br>I saw professional rescuers arriving and decided to turn away (13)<br>I received an app notification that my help was no longer required (6)<br>I couldn't find the location or the person needing CPR (3)<br>The call was determined not be a cardiac arrest and no CPR was required (2)<br>I could not access the location because of some physical barrier (1)							Why not?<br>another individual already performing CPR<br><br>EMS was already caring	Why not?<br>hands only CPR (2)<br><br>patient had signs of life<br><br>EMS was caring for patient<br><br>Another individual	Why not?<br>No AED available<br><br>Patient had signs of life<br><br>No attempt	Why not?<br>I saw an AED on my way to the emergency and brought it with me	Why not?<br>EMS already present<br><br>I was afraid of getting a contagious disease

		The address information was incomplete, or I didn't know how to get to the location provided in the alert (1)<br>I didn't feel adequately trained/prepared (1)<br>App crashed (1)	The location was a facility where I thought other trained medical staff would respond (1)<br>The alert was removed/cleared from my device prior to my arrival (1)							for patient	performing rescue breaths			

Table S1B. PulsePoint survey responses from June 2020 to September 2023. Because of the voluntary nature of the survey and the design of the survey form, the dataset is incomplete; however, all available data have been tabulated. Note: Questions 8 and 9 are related; therefore, the denominator of question 9 is based on the affirmative responses of question 8.
